# Legal gender recognition in times of change at the European Court of Human Rights

**DOI:** 10.1007/s12027-022-00710-z

**Published:** 2022-05-24

**Authors:** Lena Holzer

**Affiliations:** Graduate Institute of International and Development Studies in Geneva, Chemin Eugène-Rigot 2, 1202 Geneva, Switzerland

**Keywords:** Legal gender recognition, Transgender persons, Human rights, European Court of Human Rights

## Abstract

The COVID-19 pandemic has made the role of identity papers for the enjoyment of human rights once more obvious. It is thus a suitable moment to analyse the current implementation of the right to change the gender and/or name on official documents and civil registries in Europe. This article specifically examines the jurisprudence on the right to gender recognition of the European Court of Human Rights. It concludes that the Court is moving towards recognising the right to change one’s legal gender and/or name on an unconditional basis, and that it will need to deliberate on the right to be free from any state-imposed gender label in the future.

## Introduction

The COVID-19 pandemic has significantly heightened the number of times that people must show their identity papers in Europe. The requirement to provide identity papers alongside COVID Certificates for accessing public and private spaces in many European states has reinforced the role of identity papers as an entrance ticket for participation in society. This has had negative implications especially for people without any valid identity paper, such as undocumented migrants, and those with “incorrect” identity papers.^1^ The latter group includes certain trans and intersex persons, notably those who have a gender marker and/or a name on their identity documents that do not reflect their gender identity, gender expression and/or physical characteristics in a normative manner. Given the current context in which the role of identity papers for the enjoyment of certain human rights is once again becoming evident, this article analyses the right to change the gender marker and name on identity documents in Europe.

Whilst the effects of the pandemic’s containment measures provide an impetus to investigate states’ identity management more generally, my focus in this article lies on examining the right to change the legal gender as guaranteed by the European Court of Human Rights (ECtHR). The relevant case law on the “right to gender recognition” at the European Court of Human Rights so far concerns only the right of trans persons to change their legal gender from female to male or *vice versa*. This is also why I will focus almost exclusively on the relevance of legal gender recognition for trans persons who want to be legally recognised as a man or a woman. However, in considering the future of legal genders at the European Court of Human Rights, I will briefly discuss the right to gender recognition of intersex persons and persons who want to be legally registered with a gender other than female (F) or male (M).

The approach taken in this article is, on the one hand, a descriptive one, which exposes the legal arguments and social rationales shaping the discussion on the right to gender recognition at the European Court of Human Rights. On the other hand, I also conduct a normative analysis, proposing ways in which the right to gender recognition should develop so as to guarantee the widest possible human rights protection in the future.

The article continues with a discussion on the legal and social relevance of the right to gender recognition. I then provide an overview of current state practice in relation to gender recognition in Europe, which is followed by an analysis of the relevant European Court of Human Rights case law. In my discussion on the jurisprudence of the European Court of Human Rights, I examine how the Court initially constructed legal gender assignment as immutable and as being determined by “genitocentrism”. Subsequently, I analyse the connection between legal gender and heteronormativity in the law, as reflected in the Court’s jurisprudence. This is followed by a discussion on recent gender recognition cases which outlawed the previously supported genitocentrism and specifically concerned trans persons’ right to change their name. Lastly, I reflect on the future of legal genders at the European Court of Human Rights.

## Legal and social relevance of the right to legal gender recognition

The COVID-19 pandemic has once again made the relevance of state registration and identity documents for the enjoyment of many human rights obvious. Legal identification instruments are part of what Arendt calls “the right to have rights”, as they incorporate an individual into a community. On the basis of this, they can enjoy certain rights and entitlements.^2^ The information registered on identity papers and in state registries – such as data on a person’s nationality, age and legal gender – provides the public with an indication on the individuals’ legal status in a given community.

Recording gender in state registries and on identity papers has up to now been relevant for the functioning of legal systems, since arguably all Council of Europe states continue to have some laws that distinguish between individuals based on their legal gender.^3^ This is the case despite the fact that most states are working towards reaching formal gender equality in the law. Laws that often remain highly gendered include laws on marriage and legal partnerships, laws on military conscription^4^ or laws on social security, such as those concerning retirement ages.^5^ Sometimes it is specifically laws with the purpose of achieving substantive gender equality that rely on a distinction between individuals based on their gender. These can be laws on quotas for women in public and private leadership positions or can be equal-pay legislation.^6^ In general, the higher the number of laws making a difference between individuals based on their (legal) gender, the more relevant it is for trans persons to be able to access gender recognition in order to live their life in accordance with their gender identity.

However, the information presented on official documents and in state registries, such as gender information, does not merely indicate a person’s legal status in a community. Instead, it also influences a person’s social status. Smart has precisely analysed the “law’s ability to impose its definition of events on everyday life.”^7^ Accordingly, legal statuses reflected on official documents impose themselves on everyday life events by influencing the social status that a person enjoys in a community. Foucault and Scott similarly argue that state classification can be a major instrument in normalising and institutionalising social divisions and hierarchies.^8^ Indeed, an increasing number of studies reveal that a legal gender marker on IDs can generate a variety of negative effects on the social status that gender non-conforming persons enjoy.^9^

For example, a study on legal gender recognition conducted by the EU Commission in 2018 and 2019 analyses the various social effects of gender information on identification documents for trans persons. It shows that changing one’s legal gender has sometimes led to increased feelings of security, improved people’s access to certain services, reduced the psychological burden on them, and enabled self-recognition.^10^ Gender recognition has also had positive implications for people’s mental health and self-confidence, reducing the stress and shame experienced in identity controls. Participants in the EU survey further explained that legal gender recognition made them feel empowered, gave them the confidence to take up travelling, and increased their access to employment and education. Moreover, being recognised with one’s gender identity has sometimes prevented instances of misgendering and increased people’s acceptance among family members and social environment, which shows how legal recognition can act as an instrument of social validation. Lastly, legal gender recognition has further correlated with improvements in accessing trans-specific health care and has facilitated entry into marriage or legal partnerships.^11^

Data further discloses that discrimination against trans persons with an “incongruent” gender marker on IDs is caused not only by transphobia, but it is also mediated by other forms of discrimination, such as race and class discrimination. Whilst data on intersectional forms of discrimination experienced by trans persons in Europe is still scarce, the 2015 U.S. Transgender Survey provides an indication of how experiences of discrimination are shaped by intersecting social statuses. The Survey reveals that 2% of the 27,715 respondents had been assaulted or attacked when showing “incongruent” identity documents in the U.S. However, the number was higher for Middle Eastern (9%), “American Indian” (6%) and Black respondents (4%).^12^ The surveyed experiences also differed according to the participants’ gender identity, as 13% of trans women, 9% of men and 6% of non-binary persons were asked to leave a certain place after showing their “incongruent ID”.^13^

This data suggests that gender information on identity documents has negative effects especially for trans women and trans persons who are also racialised as non-white, creating compounded disadvantages for non-white trans women.^14^ Furthermore, the effects of gender markers are particularly detrimental for trans persons who run a high risk of being subjected to identity and security checks. These can include individuals in places administered by authoritarian or semi-authoritarian regimes, sex workers, persons in humanitarian emergencies, homeless people, migrants, racialised persons, persons accessing state benefits (*e.g.*, ration cards) and persons who are seen as belonging to certain religious or ethnic groups. As mentioned, the increased number of security checks conducted by private and public actors in the COVID-19 pandemic has put an additional burden on these already-policed trans populations.

One of the main purposes of legal gender recognition is thus to avoid the above-mentioned negative consequences of showing “incongruent” gender markers on IDs. It can prevent the forced outing of trans persons by providing them with the possibility of aligning their official documents with their gender identity, gender expression and/or gender role. However, the EU Commission has stressed that despite the fact that providing access to gender recognition can significantly improve the well-being of trans persons, it is not a silver bullet for eliminating the wide-spread prevalence of discrimination against trans persons. Easily accessible gender recognition can avoid public scrutiny of trans persons who “pass”^15^ in the gender noted on their official documents. However, those who do not pass will continue to face discrimination, violence and harassment in the public, including when showing their documents.^16^ Moreover, not only have few European states recognised non-binary legal gender categories up to now, but showing an X as a gender marker on official documents can also lead to even more discrimination than showing an M or an F.^17^

This highlights that gender recognition will alone not resolve the root cause for the discrimination and exclusion experienced by trans persons. Deeply engrained transphobia and cisnormativity^18^ in legal and social relations will need to be tackled also by other means. However, gender recognition can constitute an important instrument to make the everyday lives of trans persons easier and safer.

## European state practice of gendering legal status

As legal gender recognition can positively impact the well-being of some individuals, trans persons have started to fight for the right to change the legal gender in Europe decades ago. According to Pfäfflin, Swiss judges were the first ones in modern history to grant trans persons the right to change their legal gender status after the undertaking of surgical interventions in the 1930s.^19^ However, Sweden reportedly became the first state in the world to adopt a specific law codifying the right to change one’s legal gender in 1972.^20^ Since then, many states in Europe and elsewhere followed the Swiss and Swedish model of granting trans persons the right to change their legal gender either through court decisions or the adoption of legislation.

In an approach similar to that taken in its previous case law,^21^ the European Court of Human Rights in *X and Y v. Romania* (2021) analysed current practice in relation to the right to gender recognition of Council of Europe member states. For this purpose, it relied on the Trans Rights Map 2020 established by Transgender Europe, which showed that nowadays most Council of Europe states allow a change of legal gender and/or name, at least under certain circumstances. Only ten Council of Europe member states did not provide any clear procedure for gender recognition in 2020, whereas the rest provided, at least to adults, some sort of juridical and administrative route to changing their legal gender.^22^ Some of these routes rely on requirements that applicants must fulfil before being allowed to change their legal gender. These include the requirement of being sterilised, of having undergone gender affirmation treatment, of being diagnosed with a gender identity disorder, or of being unmarried.^23^ However, an increasing number of states – at least nine states at the time of writing (early 2022) – allow adults and sometimes also minors to change their legal gender without having to satisfy any of these conditions.^24^

The introduction of unconditional gender recognition procedures correlates with an increase in the number of persons changing their gender and/or name.^25^ This might also be due to the fact that the financial costs of changing one’s legal gender are lower in states that provide gender recognition on an unconditional basis than in states with restrictive gender recognition laws. In countries that ask individuals to fulfil intrusive medical requirements, or where requirements differ from case to case, the median cost for legal gender recognition is €814–822. This amount is far higher than the monthly minimum wage in several EU Member States. The median cost of legal gender recognition is instead about €80 in states that provide for the possibility of changing one’s legal gender without fulfilling any restrictive requirements.^26^

In fact, legal gender recognition can be quite costly, especially if the right to change one’s legal gender is dependent upon the payment of fees and the undertaking of medical and psychological consultations. These psycho-medical services can be expensive and create a disproportionate burden for persons in financially precarious situations or without access to public health care. Indeed, the EU Commission report holds that individuals facing intersectional forms of exclusion, such as non-citizens, persons with disabilities, and persons from a low socio-economic class, face additional obstacles to access gender recognition next to the general barriers created by restrictive requirements.^27^

Not only do the procedures to change legal gender vary in different states but also the possibility of changing one’s name is guaranteed differently across European states.^28^ Most people change their name alongside changing their legal gender, since most first names and some last names are gendered and can thus indicate a person’s legal gender. This is especially the case in states where it is mandatory to have a name that is gendered in the same binary manner as one’s legal gender. For example, Hungarian law requires parents to give their child a first name that corresponds to their legal gender,^29^ whilst the same is true for Germany, with the exception that Germany allows parents to assign a gender-neutral first name.^30^ In some countries, such as Greece and Poland, last names can be gendered, mostly through the addition of a gender-specific suffix to the name (*e.g.*, Pappas/Papadopoulos, Kowalski/Kowalska).^31^

The discussion on names shows that gender markers (*i.e.*, F, M or X) are not the only means to present gender information on official documents. Instead, names, titles (*e.g.*, Mr/Ms/Mrs, M/Mme/Mlle) and personal identity codes on IDs can also transmit information on a person’s legal gender. In fact, it is mostly the names reflected on COVID Certificates that create problems for trans persons when having to show these documents during the on-going pandemic. Moreover, many states in the Council of Europe, especially Eastern European and Scandinavian states, issue personal identity codes for social security and taxation purposes that reflect a person’s legal gender.^32^ For instance, the Czech Identity Number, which is shown on the national identity card and needed for most official actions in the Czech Republic, contains two specific digits that express a person’s legal gender. If these specific digits are within the range of 01–12, the person concerned is legally considered male, but if they are within the range of 51–62, the person’s legal gender is female.^33^ Reflection on these gendered personal identity codes makes it clear that the right to gender recognition entails being able to change *any* information on official documents and in civil registries that can indicate the status of a person’s legal gender.

## A long journey in Strasbourg…

The fact that most Council of Europe member states nowadays provide a gender recognition procedure has also been induced by relevant judgments of the European Court of Human Rights. For instance, both France and the United Kingdom introduced the possibility of changing one’s legal gender only after having lost a case on this matter at the European Court of Human Rights.^34^ The Court was for the first time asked to rule on the question whether the European Convention on Human Rights (ECHR) ensures the right to change one’s legal gender in *Rees v. The United Kingdom* (1986).^35^ Since then, it has delivered numerous judgments on the right to gender recognition, some of which are discussed in this article.

### The immutability of the legal gender assignment

In *Rees v. The United Kingdom*, the applicant claimed that denying him the right to change the legal gender on his UK birth certificate constituted a violation of Article 8 ECHR, which ensures the right to a private and family life. The Court ruled against the applicant in this case and found no positive obligation that would require the UK to ensure the possibility of changing legal gender on a birth certificate. It concluded that it was within the UK’s margin of appreciation to decide on the balance between an individual’s rights to private life and public interests, because the requested changes in the birth registry “would have important administrative consequences and would impose new duties on the rest of the population.”^36^

An argument put forward by the UK in *Rees* was that the information on birth certificates must be immutable as they reveal “historical facts”. These facts were needed “to enable the establishment of the connections of families for purposes related to succession, legitimate descent and distribution of property.”^37^ This argument used by the UK government to justify the denial of gender recognition implies that there are laws that make a difference based on people’s gender in establishing the right to inherit, receive and own property. In the context of the 1980s, the laws in question were laws that discriminated against women in terms of their access to property. Thus, gender discriminatory laws are based on the use of gender as a stable legal identity characteristic.

The Court accepted the argument offered by the UK in this case and similarly referred to the need to ensure succession, legitimate descent, and distribution of property as justification for denying an individual the right to change the gender on the birth certificate.^38^ This is noteworthy since, at the time of the proceedings, the UK had repealed most laws that categorically discriminated against women in terms of their access to property.^39^ Even though the reasoning brought forward by the UK was not entirely consistent, it shows that an underlying reason for the existence of legal genders is the existence of laws making a difference based on gender. Historically, these laws often discriminated against women, but, as discussed above, they nowadays also include gender equality laws that aim to reverse historically established structures of inequality.

### Grounding legal gender assignment in genitocentrism

The *Rees* case was followed by multiple other cases concerning the right to gender recognition at the European Court of Human Rights. Trans communities in Europe had an interim success in *B. v. France* (1992), where the Court found that Article 8 ECHR created a positive obligation on France to allow the applicant to change their legal gender on at least some official documents.^40^ However, it was only in *Christine Goodwin v. The United Kingdom* (2002)^41^ that the Court unequivocally recognised an applicant’s right to change the legal gender on her birth certificate and thus for all legal purposes (*i.e.*, marriage). It found that the state had no longer struck a fair balance between the applicant’s right to private life and the public interest of avoiding any major bureaucratic changes to the birth registration system that could affect “access to records, family law, affiliation, inheritance, criminal justice, employment, social security and insurance.”^42^

The fact that the applicant, Goodwin, had undertaken gender affirmation treatment considerably affected the Court in its decision in the *Goodwin* case. The Court argued that since Goodwin had modified her body appearance through gender affirmation surgeries, which were paid for by the UK National Health Service, she must have the right to change legal gender so as to ensure “coherence of the administrative and legal practices.”^43^ This reasoning relied on the logic that persons with a normatively looking “feminine” body appearance must also be legally recognised as women.

Gonzalez-Salzberg argues that the Court’s focus on the applicant’s body appearance for establishing her right to gender recognition represents an example of “genitocentrism”.^44^ This term was coined by Sharpe to denote the common assumption that the appearance of a person’s genitals determines a person’s legal gender.^45^ Whilst the *Goodwin case* was thus a milestone in the recognition of the right to gender recognition in Europe, it reproduced the idea grounded in genitocentrism that a person’s legal gender must correspond to an individual’s physical appearance in a cisnormative manner. The tailoring of the Court’s decision to the circumstances of the applicant, who had undertaken gender affirmation treatment, further meant that the *Goodwin* judgment did not prevent states from making gender affirmation surgery a precondition for allowing a change of legal genders.

### Protecting heteronormative laws through legal gender

The *Goodwin* case presented not only an example of genitocentrism but also exposed the relationship between legal gender recognition and heteronormativity in the law. In addition to recognising the right to change one’s legal gender, the Court held that Article 12 of the European Convention on Human Rights ensured Goodwin’s right to marry her partner, a legally recognised man.^46^ In previous case law, the Court had allowed states to deny trans persons to marry somebody with a legal gender different from their gender identity, alongside denying them the right to gender recognition.^47^ The crux behind these previous decisions was that if the applicants had been allowed to marry their partner with an opposite gender identity but with the same legal gender, their marriage would have legally been a homosexual one as long as they could not change their own legal gender. The Court thus needed to allow states to deny the applicants the right to marry their partners in order to create coherence with its stance that states are under no obligation to recognise same-sex marriage, as explicitly stated in other cases.^48^ However, in *Goodwin*, the Court reasoned that once Goodwin would change her legal gender, marrying a person legally recognised as man would no longer constitute a homosexual marriage. The marriage must thus be permitted by the law.

The role of heteronormativity in establishing the right to gender recognition manifested in the *Goodwin* case is also reflected in the Court’s judgment in *Hämäläinen v. Finland (2014)*. In this case, the Court held that Finland had not violated Article 8 ECHR by requiring a married trans persons to get divorced or to change their marriage to a registered partnership before allowing them to change their legal gender. At the time of the proceedings, Finland had not yet recognised same-sex marriage, which meant that it did not approve the change of legal gender of a married person since this would render their marriage legally a homosexual one.^49^

The Court’s reasoning in *Goodwin* and *Hämäläinen* exposed what some scholars and judges have argued, namely that legal gender assignment has functioned as a tool to safeguard marriage as a heterosexual union.^50^ The EU Commission has held similarly that the requirement to be unmarried for legal gender recognition as upheld in *Hämäläinen* “represent[s] society’s primary interest to enforce the binary gender system.”^51^ Already in *Rees* in 1986, the Court referred to the institution of marriage as a means to demonstrate the need to have a proof of one’s sex assigned at birth, since “marriage is defined as a voluntary union for life of one man and one woman to the exclusion of all others.”^52^ This shows the role of the legal gender assignment to individuals at birth in protecting heteronormativity in the law.

### Outlawing genitocentrism

Whilst the *Goodwin* case served for a long time as the standard on legal gender recognition set by the European Court of Human Rights, recent cases have overruled some of the decisions reached in Goodwin. In fact, the genitocentrism that was represented in the Goodwin case is no longer reflected as such in the Court’s current interpretation of the ECHR. Instead, the Court’s judgments outlawed the requirement to undergo gender affirmation treatment as a precondition for accessing gender recognition in Council of Europe member states in *A.P.*, *Garçon and Nicot v. France (2017)* and *X and Y v. Romania (2021)*.

In *A.P.*, *Garçon and Nicot v. France*, the Court held that requiring individuals to undertake sterilisation or any medical procedure that likely results in sterilisation before allowing them to access gender recognition constitutes a violation of Article 8 ECHR. This represents an important decision for safeguarding the right to bodily integrity of trans persons. However, the Court also ruled that asking individuals to be diagnosed with a gender identity disorder, mostly called “gender dysphoria”, and/or to undergo medical examinations does not violate the ECHR.^53^

The more recent decision in *X and Y v. Romania* goes further by holding that states can no longer require individuals to undertake “gender reassignment surgery” before allowing them to change the legal gender.^54^ It thus departed from its previous positions which reflected the genitocentric assumption that the appearance of genitals determines one’s legal gender assignment. It found that Romania did not struck a fair balance between the applicants’ rights to private life and the public interest of safeguarding legal certainty and inalienability, consistency and reliability of civil status.^55^ This decision supports the vision provided by the Council of Europe Parliamentary Assembly, which, in a resolution in 2015, called on member states to “develop quick, transparent and accessible procedures, based on self-determination, for changing the name and registered sex of transgender people.”^56^ However, aligning the jurisprudence of the European Court of Human Rights entirely with this vision requires that the Court further outlaws other requirements for gender recognition, such as the condition of being diagnosed with a gender identity disorder or of being unmarried.

Even though the European Court of Human Rights has still to declare certain requirements for accessing gender recognition to be contrary to the ECHR, it has already clarified that states cannot categorically deny the right to gender recognition to non-nationals. In *Rana v. Hungary (2020)*, the Court recognised that Hungary had violated Article 8 ECHR by denying a refugee, who could not change his legal gender in his country of origin, the right to access the Hungarian gender recognition procedure.^57^ This decision thus makes clear that immigration status cannot be used so as to exclude categorically certain individuals from changing their legal gender in their country of residence.

However, whilst the trans communities have had many legal victories in the last two decades, the recent case *Y. v. Poland (2022)* draws attention to the fact that various aspects of the right to gender recognition are far from being settled in European human rights law. In this case, the claimant had changed his legal gender, and such change was merely added as annotation to his original birth certificate. The authorities refused issuing a new birth certificate that noted his current legal gender. As a consequence, the claimant alleged a violation of Article 8 and 14 of the European Convention on Human Rights due to the forced outing of his trans history through the annotation on his birth certificate.^58^

The Court sided with Poland by holding that mentioning a change of legal gender on a person’s birth certificate was not contrary to the Convention. It justified its decision by holding that the birth certificate can be mostly substituted by other documents that do not reflect a change of legal gender, including a short extract of the birth certificate.^59^ Moreover, despite recognising that annotating a change of legal gender on the birth certificate “could cause the person concerned to experience some distress,”^60^ the Court found that knowing a person’s gender assigned at birth could be in “public interests” in certain circumstances. As Cannoot points out, this reflects the “stereotyped, cisnormative logic of the official sex/gender registration system,”^61^ in which identifying with the gender assigned at birth remains the *status quo* and trans persons are rendered the exception by making their trans history legally visible.

### Disconnecting legal gender and names

Whilst the jurisprudence on gender recognition of the European Court of Human Rights focuses mostly on changes of gender markers, the Court addressed the right to change one’s first name explicitly in *S.V. v. Italy (2018)*. In this case, it held that Italy had violated Article 8 ECHR by denying the applicant a change of name before she had modified her legal gender. Changing one’s legal gender on official papers and civil registries in Italy was at that time possible only after having undertaken gender affirmation surgery and having obtained a court order. According to the European Court of Human Rights, during this lengthy procedure for changing the applicant’s legal gender, Italy had a positive obligation to allow her a change of first name.^62^

The *S.V.* case recognised that states must not *categorically* deny the possibility of carrying a first name that does not correspond to a person’s legal gender in a binary cisnormative manner. However, it is important to note that the Court’s decision in *S.V. v. Italy* was a response to the specific circumstances of the case. The Court took the view that the Rome District Court had violated the applicant’s right to a private life because it had not taken into account the applicant’s concrete situation when refusing to allow her a change of name. S.V. had started to transition several years before, and her physical appearance and social role had long identified her as a woman. Given these circumstances, the Court held that it was disproportionate to delay a change of the applicant’s name for two and a half years until she had undergone gender affirmation treatment and consequently changed her legal gender. It was “the rigid nature of the judicial procedure for recognising the gender identity of transgender persons [that] had left S.V. for an unreasonable period of time in an anomalous position apt to engender feelings of vulnerability, humiliation and anxiety.”^63^

Despite the fact that the European Court of Human Rights’ decision in *S.V. v. Italy* responds to a context-specific case, it sets an important legal precedent for trans persons’ right to change their name in Europe.^64^ Being able to erase one’s “deadname”^65^ on official documents and in civil registries is especially relevant in situations when trans persons want or need to use documents that do not carry a gender marker or that cannot be modified after legal transition. For example, many university diplomas do not contain a gender marker, but the name mentioned on the diploma can provide an indication of a person’s legal gender. These diplomas are sometimes unchangeable after they are issued, which means that showing a person’s deadname on them can cause forced outings in the future, especially in professional contexts when diplomas have to be shown.^66^

Moreover, most drivers’ licences in Europe and a few personal identity cards, such as the German or Greek personal identity cards, do not carry any gender marker. The same is mostly true for COVID Certificates, which rarely show a legal gender but do mention first and last names. Changing the name on these documents allows trans persons to use them as legal proofs of identity while undergoing a change of legal gender in the civil registry. Whereas modifying legal gender can affect a person’s rights and duties (since some laws continue to make a difference based on gender), changing a name rarely modifies a person’s legal status. Changing a name is also quite common, considering that most marriages result in the adoption of a new surname by one person. It thus seems questionable whether refusing a change of name to trans persons is proportionate to the purpose of ensuring an accurate population registration, safeguarding personal identification, and linking the individual concerned to a family, as proposed in *S.V. v. Italy*.^67^

## The future of legal gender at the European Court of Human Rights

The foregoing discussion has shown that the approach of the European Court of Human Rights to addressing the right to gender recognition has changed significantly since 1986. The Court is currently moving towards endorsing unconditional gender recognition laws, which allow an individual to change their legal gender and/or name without fulfilling any restrictive requirements. However, the recent decision in *Y. v. Poland* shows that cisnormativity is still deeply in engrained in legal systems and judges’ conception of appropriate gender norms in Europe.^68^ Moreover, certain European states, such as Hungary, have recently implemented laws that curtail the right to gender recognition and clearly go against the jurisprudence of the European Court of Human Rights.^69^

The saga of trans persons’ right to be legally recognised with their gender identity and their name on an equal basis with cis persons is far from over. Not only are several cases concerning the right to gender recognition currently pending at the European Court of Human Rights, but also social and legal perspectives on the legitimacy of using gender as a (binary) identity characteristic are constantly evolving. This is shown by the fact that an increasing number of Council of Europe states are adopting unconditional gender recognition laws and/or recognise non-binary legal gender categories. Moreover, the question of why states continue to make categorical gender differences between humans by registering a legal gender at birth is increasingly debated among scholars and law-makers in Europe.^70^

In the future, the European Court of Human Rights will likely be asked to re-address the legality of the so-called “divorce requirement”, as upheld in the *Hämäläinen* case. Moreover, it will certainly be confronted with the question of whether the highly prevalent state practice of asking individuals to be diagnosed with a gender identity disorder before allowing them to change their legal gender is proportionate to the public interest of ensuring the stability of legal status. Another question likely to be debated by the Court in the future is whether requiring judicial proceedings, in the form of having to ask courts for permission to change one’s legal gender, instead of going through an administrative procedure, is in accordance with the ECHR. In addition, the Court is expected to provide its decision in the pending case of *Y v. France* in the near future. This case is the first one at the European Court of Human Rights that concerns an intersex person who is claiming the right to change their legal gender to “neutral” or “intersex” in the French civil registry.^71^

The future of legal gender at the European Court of Human Rights will, however, not only depend on the different subject matters decided by the Court but also on *how* the Court approaches the issue of gender recognition. Until now, the Court has treated issues of gender recognition as exclusively falling within the ambit of Article 8 ECHR. The applicants in relevant cases have always invoked the right to private life (Article 8), but in some cases, they have additionally also claimed a violation of the right to be free from torture, degrading and inhumane treatment (Article 3)^72^ and the right to non-discrimination (Article 14).^73^ Nevertheless, the Court has largely ignored claims brought under Article 3 and 14 when discussing the right to gender recognition.

I see great value in treating the denial of gender recognition as violating the right to be free from torture, degrading and inhuman treatment, an approach also suggested by Bassetti.^74^ This could draw attention to the violent effects generated by legal misrecognition. Moreover, discussing issues of gender recognition as part of the right to non-discrimination would highlight that trans persons’ gender identities are currently not recognised on an equal basis with cis persons’ gender identities. This would help to dismantle the cisnormativity currently codified in the law and ensure equality, especially in the global health crisis where identity papers are highly relevant in accessing basic services, entitlements and spaces.

However, an even more effective tool to weaken cisnormativity in the law could be to address issues of legal gender recognition as ensuing negative state obligations. So far, the European Court of Human Rights has treated the right to gender recognition as being part of the positive state obligations deriving from Article 8. It has reasoned that states have a positive duty to implement procedures that allow modifying the legal gender. However, addressing the right to gender recognition from the perspective of negative state obligations could shift the question to whether states must *abstain* from assigning a gender to individuals. In fact, this would fit the Court’s common approach, since the right to private and family life is paradigmatic for engendering negative obligations that require states to refrain from interfering into a person’s life.^75^ This also highlights that the use of gender as a (binary) identity characteristic assigned to individuals is part of a general discussion on how far states can interfere in people’s lives in their population management and surveillance.

The latest point further raises the question of whether the right to gender recognition in the future will also include being free of any state-assigned gender label. My discussion of the *Rees* case highlighted that legal gender assignments have historically played a strong role in ensuring the categorical exclusion of women from certain (property) rights. Moreover, the European Court of Human Rights’ jurisprudence reflects the interrelationship between assigning a legal gender to individuals and establishing heteronormativity in the law. Hence, other than codifying heterosexism in the law, what purposes exist for using gender as a legal identity characteristic? Why do states continue to assign a legal gender to individuals at birth and on other instances in life?

These questions are complicated ones and answering them requires having context-specific understandings of social and legal relations. Responses to these questions will need to take into account the utility of relying on legal gender categories for reversing historical patterns of structural inequality, such as through gender equality laws, like equal pay legislation. In any case, the European Court of Human Rights will likely have to deliberate on the necessity and proportionality of assigning a legal gender to individuals in the future since an increasing number of individuals in Europe seek to live without any state-assigned gender label.^76^ The future of legal gender at the European Court of Human Rights is thus uncertain and open to be constructed in a way that destabilises cisnormativity and heterosexism in social norms and laws in Europe.

## Footnotes

1 ‘COVID-19 Certificates and Undocumented Migrants: For Some, the Risk Is More Exclusion’ (*PICUM*, 18.1.2022) <https://picum.org/covid-19-certificates-undocumented-migrants/> accessed 9.3.2022.

2 *Arendt* [2].

3 Dutch researchers have found that there “remain many provisions in which gender somehow plays a role” in the Netherlands, a state with a high degree of formal gender equality. See: *van den Brink, Tigchelaar* [5], p. 5–6.

4 For an overview of states practicing mandatory conscription and their respective gender policies, please see: ‘Military Service Age and Obligation’ (*CIA – The World Factbook*) <https://www.cia.gov/the-world-factbook/field/military-service-age-and-obligation/> accessed 15.2.2022.

5 Retirement ages remain different for women and men in many Council of Europe Member States. In *Andrle v. the Czech Republic (2011)*, the ECtHR found that having lower retirement ages for women than for men does not violate the ECHR as long as they serve the purpose of compensating for the double burdens faced by women in the Czech Republic. However, EU Gender Equality Directives (*i.e.* Directive 2006/54/EC and Directive 79/7/EEC) make gender-based difference in access to social security, such as retirement, no longer legal in EU Member States.

6 For example, companies with more than 100 employees in Switzerland must conduct an internal equal pay analysis every year, analysing the difference in salaries between women and men. This requires making a distinction between employees based on their gender. See: 151.1 Federal Act on Gender Equality (Gender Equality Act, GEA) 1995 Art. 13.

7 *Smart* [26], p. 4.

8 *Foucault* [13], p. 140–142; *Foucault* [14], p. 242; *Scott* [24], p. 82–83.

9 For example: *European Commission* [8]; *James et al.* [19].

10 *European Commission* [8], p. 10.

11 *Ibid.*, 173–187.

12 *James et al.* [19], p. 90.

13 *Ibid.*

14 As reported by Human Rights Watch, trans persons of colour face a significantly higher risk of experiencing violence in the U.S. than white trans persons. Reportedly, three-quarters of all trans persons killed in the U.S. in 2020 were people of colour, with black trans women having the highest likelihood of experiencing violence. See: *Human Rights Watch* [18], p. 2.

15 I use the term “passing” to refer to situations in which people are perceived as their gender (identity) and without any history of gender transition. For trans persons, passing means that they are read as cis persons.

16 *European Commission* [8], p. 14.

17 *Nationale Ethikkommission im Bereich der Humanmedizin NEK* [21], p. 23; ‘Intersexuelle Menschen: Warum in Hessen nur wenige ihren Geschlechtseintrag in “divers” ändern lassen’ *Frankfurter Allgemeine* (17.11.2019) <https://www.faz.net/1.6489647> accessed 14.2.2021.

18 The term cisnormativity refers to the fact that being cis is considered as “the norm”, while being trans or gender non-conforming are constructed as “the exception”.

19 *Pfäfflin* [22], p. 17. Historical records show that even before the twentieth century, many states granted individuals the right to change the legal gender on *ad hoc* basis. However, these individuals were mostly intersex persons whose bodies developed in a manner that was considered incongruent with the gender assigned at birth. See: *Houbre* [17], p. 10; *Foucault* [12]; *Wijffels* [30], p. 195–196.

20 *Scherpe* [23], p. 17.

21 *AP, Garçon and Nicot v France* [2017] European Court of Human Rights Applications 79885/12, 52471/13, 52596/13 [70–72].

22 *X et Y c Roumanie* [2021] Cour européenne des droits de l’homme Requêtes 2145/16 et 20607/16 [84–88].

23 The Trans Rights Map 2021 provides information on the requirements for legal gender recognition in each Council of Europe member state. See: TGEU, ‘Trans Rights Map 2021. Europe and Central Asia’ (2021) <https://tgeu.org/wp-content/uploads/2021/05/tgeu-trans-rights-map-2021-index-en.pdf> accessed 27.1.2022.

24 In early 2022, Belgium, Denmark, Iceland, Ireland, Luxembourg, Malta, Norway, Portugal and Switzerland allow a change of legal gender without any major restrictive requirements.

25 *European Commission* [8], p. 9.

26 *Ibid.*, 13.

27 *Ibid.*, 12.

28 For an overview of the procedures for changing one’s name in Europe, see *Chiam et al.* [6], p. 110–175.

29 The lists of approved female and male names in Hungary can be accessed at: http://www.nytud.hu/oszt/nyelvmuvelo/utonevek/osszesffi.pdf (accessed on 27 Jan 2022) and http://www.nytud.hu/oszt/nyelvmuvelo/utonevek/osszesnoi.pdf (accessed on 27 Jan 2022).

30 Germany, Allgemeine Verwaltungsvorschrift zum Gesetz über die Änderung von Familiennamen und Vornamen 1980 [NamÄndVwV] para 67; *1 BvR 576/07*.

31 *Walkowiak* [29], p. 223; *Lawson* [20], p. 178.

32 For an overview of legal gender recognition procedures in European countries, please see *Chiam et al.* [6], p. 110–175. See, for example, also *Swedish Tax Agency* [27], p. 11; Finnish Digital and population data services agency, ‘Personal Identity Code’ <https://dvv.fi/en/personal-identity-code> accessed 14.2.2022; Republic of Estonia, ‘How to Use Your Digital ID’ (*E-Residency, Knowledge Base*) <https://learn.e-resident.gov.ee/hc/en-us/articles/360000624498-How-to-use-your-digital-ID> accessed 14.2.2022.

33 133/2000 Coll. Act on Population Registration and Birth Numbers and on Amendments to Certain Acts (Population Registration Act) Title III, Section 13 (3).

34 Following the decision in *B v. France (1992)* at the ECtHR, the French Cour de Cassation recognised a trans person’s right to change their gender in the French civil registry. Similarly, the UK adopted the Gender Recognition Act in 2004 after the ECtHR’s decision in *Christine Goodwin v. the United Kingdom (2002)*. See: *B v France* [1992] European Court of Human Rights Application 13343/87; *9111 900* (Cour de cassation); *Christine Goodwin v The United Kingdom* [2002] European Court of Human Rights Application 28957/95; Gender Recognition Act 2004.

35 *Van Oosterwijck v. Belgium (1980)* also concerned the right to gender recognition. However, the Court never ruled on the merits of the case as it found the application inadmissible.

36 *Rees v The United Kingdom* [1986] European Court of Human Rights Application 9532/81 [42(a)].

37 *Ibid.*, 21.

38 *Ibid.* 42(b), 43.

39 An exception in this regard is the principle of “the presumption of advancement” established in English law. This principle makes a gender-based difference as the transfer of property from a wife to her husband is treated in a different manner from the one from a husband to their wife. For more information, see: *Blackham* [4], p. 786.

40 *B v France* [1992] European Court of Human Rights Application 13343/87.

41 The *Goodwin* case was decided on the same day as *I. v. the United Kingdom (2002)*, treating the same subject matter as the *Goodwin* case. See: *I v The United Kingdom* [2002] European Court of Human Rights Application 25680/94.

42 *Christine Goodwin v The United Kingdom* [2002] European Court of Human Rights Application 28957/95 para 91.

43 *Ibid.*, 78.

44 *Sharpe* [25], p. 56.

45 *Gonzalez-Salzberg* [16] 49.

46 *Christine Goodwin v The United Kingdom* [2002] European Court of Human Rights Application 28957/95 para 101.

47 *Rees v The United Kingdom* [1986] European Court of Human Rights Application 9532/81 para 49; *Cossey v The United Kingdom* [1990] European Court of Human Rights Application 10843/84 [43]; *Sheffield and Horsham v the United Kingdom* [1998] European Court of Human Rights Applications 31–32/1997/815–816/1018–1019 [66].

48 On same-sex marriage, see also *Schalk and Kopf v Austria* [2010] European Court of Human Rights Application 30141/04; *Oliari and Others v Italy* [2015] European Court of Human Rights Applications 18766/11 and 36030/11; *Chapin et Charpentier v France*, [2016] European Court of Human Rights Application 40183/07.

49 *Hämäläinen v Finland* [2014] European Court of Human Rights Application 37359/09.

50 *Clarke* [7], p. 652; *NSW Registrar of Births, Deaths and Marriages v Norrie* [2014] High Court of Australia S273/2013 [42]; *Gonzalez Salzberg* [15], p. 173.

51 *Agius, Tobler* [1], p. 19.

52 *Rees v The United Kingdom* [1986] European Court of Human Rights Application 9532/81 para 26.

53 *AP, Garçon and Nicot v France* [2017] European Court of Human Rights Applications 79885/12, 52471/13, 52596/13 (n 21).

54 *X et Y c Roumanie* [2021] Cour européenne des droits de l’homme Requêtes 2145/16 et 20607/16.

55 *Ibid.*, 158, 164.

56 Council of Europe Parliamentary Assembly, ‘Resolution 2048 (2015), Discrimination against Transgender People in Europe. Doc. 13742’ (22.4.2015) para 6(2).

57 *Rana v Hungary* [2020] European Court of Human Rights Application no. 40888/17.

58 *Y v Poland* [2022] European Court of Human Rights Application no. 74131/14.

59 *Ibid.*, para 78.

60 *Ibid.*, para 79.

61 Pieter Cannoot, ‘Y. v. Poland: ECtHR Case Law on Gender Recognition Remains Embedded in Cisnormativity’ (*Strasbourg Observers*, 07.04.2022) <https://strasbourgobservers.com/2022/04/07/y-v-poland-ecthr-case-law-on-gender-recognition-remains-embedded-in-cisnormativity/> accessed 08.04.2022.

62 *SV c Italie* [2018] Cour européenne des droits de l’homme Requête no 55216/08.

63 *European Court of Human Rights* [9], p. 3.

64 Even though the *S.V.* case concerned the change of a person’s first name, drawing the analogy to gendered last names suggests that the Court would also find a violation of the ECHR if a state categorically denied a change of a gendered last name.

65 The name assigned to trans persons at birth, which is no longer used after their transition, is commonly called “deadname”.

66 *European Commission* [8], p. 182.

67 *SV c Italie* [2018] Cour européenne des droits de l’homme Requête no 55216/08 para 61.

68 *Y v Poland* [2022] European Court of Human Rights Application no. 74131/14.

69 Hungarian Omnibus Bill. T/9934 2020.

70 See *e.g.*, *1 BvR 2019/16* (Bundesverfassungsgericht) [65]; *Nationale Ethikkommission im Bereich der Humanmedizin NEK* [21], p. 25–26.

71 *Y v France* (*pending*) [2017] European Court of Human Rights Application 76888/17.

72 See *e.g.*, *L v Lithuania* [2011] European Court of Human Rights Application 27527/03 para 37; *X et Y c Roumanie* [2021] Cour européenne des droits de l’homme Requêtes 2145/16 et 20607/16 para 103.

73 See *e.g.*, *Y v Poland* [2022] European Court of Human Rights Application no. 74131/14 para 85; *X et Y c Roumanie* [2021] Cour européenne des droits de l’homme Requêtes 2145/16 et 20607/16 para 169; *L v Lithuania* [2011] European Court of Human Rights Application 27527/03 para 65; *Hämäläinen v Finland* [2014] European Court of Human Rights Application 37359/09 para 98; *Christine Goodwin v The United Kingdom* [2002] European Court of Human Rights Application 28957/95 para 105.

74 *Bassetti* [3], p. 291.

75 *European Court of Human Rights* [10], p. 8.

76 As the EU Commission holds, a survey by the Fundamental Rights Agency released in 2019 holds that 25% of all trans and intersex respondents consider the mentioning of sex on identity cards a problem. Moreover, a survey conducted in the UK shows that 41% of 895 non-binary persons surveyed are in favour of the elimination of public gender registration altogether. See: *European Union Agency for Fundamental Rights* [11] 33; *Valentine* [28], p. 73.
